# Age-related differences in injury pattern and hospital stay after paediatric trauma

**DOI:** 10.1186/1757-7241-20-S1-P2

**Published:** 2012-03-22

**Authors:** HQ Do, J Steinmetz, LS Rasmussen

**Affiliations:** 1 Department of Anaesthesia, Centre of Head and Orthopaedics, Copenhagen University Hospital, Rigshospitalet, Denmark; 2 Trauma Centre & Acute Admissions, Centre of Head and Orthopaedics, Copenhagen University Hospital, Rigshospitalet, Denmark

## Introduction

It is estimated that children account for around 10% of all trauma admissions. While few studies describe paediatric trauma, only one British study has described age-related differences after paediatric trauma [1].

## Aim

To describe age-related differences in paediatric trauma patients admitted to a Danish level 1 trauma center.

## Methods

We included 331 paediatric trauma patients admitted during the 9-year period 1999-2007. Subjects were studied with respect to four age groups (<1 year [n=20], 1-5 years [n=119], 6-10 years [n=72], and 11-15 years [n=120]). We analysed the groups with regard to injury type and mechanism, injury severity score (ISS), and length of stay. p<0.05 was considered statistically significant.

## Results

Injury type and mechanism were significantly different between the four groups (p<0.0001). The leading cause of trauma were burn injuries (65%) in patients aged less than 1 year, burn injuries (49%) and blunt trauma (49%) in children between 1-5 years, and blunt trauma (81%/82%) in 6-10 and 11-15 year old children. Mechanisms of blunt trauma were mainly road traffic accidents, followed by fall accidents. In total, eight (2%) patients had penetrating trauma.

ISS increased with age, however not significantly (p=0.19), median ISS 9, 9, 10, and 11 respectively.

With increasing age, a significant decline in total length of stay was observed, median 20, 15, 9, and 9 days respectively (p=0.005). However, patients aged 6 or more had a significantly longer stay at the ICU, median 0, 0, 1, and 1 day respectively (p=0.002).

## Conclusions

Burn injuries were the leading cause of trauma in children aged less than 1 year, while burn injuries and blunt trauma occurred equally in 1-5 year old children. Children older than 6 years were more often implicated in blunt trauma, predominantly road traffic accidents and fall accidents. This age-related difference in injury pattern was reflected in the total length of stay that decreased with advancing age.
